# The Secret Life of Oilbirds: New Insights into the Movement Ecology of a Unique Avian Frugivore

**DOI:** 10.1371/journal.pone.0008264

**Published:** 2009-12-16

**Authors:** Richard A. Holland, Martin Wikelski, Franz Kümmeth, Carlos Bosque

**Affiliations:** 1 Department of Migration and Immuno-Ecology, Max-Planck Institute for Ornithology, Radolfzell, Germany; 2 e-obs GmbH, Munich, Germany; 3 Departamento de Biología de Organismos, Universidad Simon Boíivar, Caracas, Venezuela; University of Oxford, United Kingdom

## Abstract

**Background:**

*Steatornis caripensis* (the oilbird) is a very unusual bird. It supposedly never sees daylight, roosting in huge aggregations in caves during the day and bringing back fruit to the cave at night. As a consequence a large number of the seeds from the fruit they feed upon germinate in the cave and spoil.

**Methodology/Principal Findings:**

Here we use newly developed GPS/acceleration loggers with remote UHF readout to show that several assumptions about the behaviour of *Steatornis caripensis* need to be revised. On average, they spend only every 3^rd^ day in a cave, individuals spent most days sitting quietly in trees in the rainforest where they regurgitate seeds.

**Conclusions/Significance:**

This provides new data on the extent of seed dispersal and the movement ecology of *Steatornis caripensis*. It suggests that *Steatornis caripensis* is perhaps the most important long-distance seed disperser in Neotropical forests. We also show that colony-living comes with high activity costs to individuals.

## Introduction

One of the most crucial challenges for biologists in the next decade is the understanding of the ecological and evolutionary processes involved in the movement of organisms [1]. This assumes particular significance given the emergence of problems with habitat fragmentation and climate change. The recently defined paradigm of “Movement Ecology” [2] calls for a need for new data not just on sequential positions in space but also on the physiological and/or behavioural state of the organism in order to fully understand why and how they move. Understanding movement ecology assumes particular significance in plants, whose seeds are dispersed by animals in fragmented habitats where avian seed dispersers play a crucial role in the ecosystem [3], [4], [5]. Despite this, high resolution data on the impact of avian seed dispersers are lacking in most cases. Seed dispersal is one of the most important processes in any ecosystem, particularly during times when anthropogenic influences fragment landscapes into small, potentially non-connected habitats [4], [5], [6], [7]. Understanding which animals provide the ecosystem services of dispersing seeds between fragments and over large distances is a major research goal for ecology [4], [5], [6], [7], [8]. Seed dispersal is particularly important in tropical forest ecosystems that suffer considerably under anthropogenic stress [9]. Animals that provide connectivity in fragments of tropical forests should be important targets of conservation [8]. Thus the aim of this study was to better understand the ecological role *Steatornis caripensis* (the oilbird) in Neotropical forests using high resolution GPS and accelerometry [10], [11].

GPS tracking technology has the possibility to revolutionise the study of the behaviour and ecology of animals in a natural setting [12], but so far its use on wild birds has been relatively limited, due to the necessity of recovering the logging device or of using expensive remote download through a satellite platform to access the data. The majority of studies have been performed on sea birds that in addition to being large, have a nesting behaviour that makes recovery of the logging device relatively easy (e.g. [13]). Amongst terrestrial birds, only the domesticated homing pigeon has been tracked in high resolution, for purposes of navigational study [14], and no wild, terrestrial central place foraging bird has yet been tracked by GPS to our knowledge. The development of a GPS device which allows data to be downloaded remotely by a UHF radio link, a far less expensive option than satellite download and available in extreme environments such as deep caves, provides a third way in which animals that are not easily re-caught can be studied in the wild with GPS precision. The addition of an accelerometer allows behavioural changes of the animal to be studied without direct observation [10], [11] and has the potential to reveal much about the movement ecology of wide ranging mobile animals that are difficult to observe directly. This device is particularly suited to the study of central place foragers which can reliably be detected at a known roosting site.

*Steatornis caripensis* [15] is a unique avian frugivore. They sally for fruit at night instead of hunting for insects on the wing, as other members of the Caprimulgiformes, such as nightjars do [16], [17]. During the day they roost in deep caves into most of which sunlight does not penetrate. The eyes of *Steatornis caripensis* have the highest light-gathering capacity of any terrestrial vertebrate, perhaps the maximum that is achievable [18], with a rod∶cone ratio of 123∶1 [19] and a density of 1 million rods/mm^2^. This, and their above described well-known nocturnal foraging habits [17], had researchers firmly convinced that *Steatornis caripensis* “…never see direct sun light” as they are “…well equipped for cavernicolous and nocturnal habits” [19]. Despite this assumption, reports exist that *Steatornis caripensis* are occasionally seen roosting during the daytime in canopy trees [20], [21] *Steatornis caripensis* are known to carry seeds over large distances back to their respective caves [15], [17], [22], [23]. The high lipid content of the fruit pulps that they consume requires lengthy gut transit times [24], although seeds can be regurgitated more quickly. Nevertheless, *Steatornis caripensis* have been seen regurgitating seeds in caves long after returning to roost there for the day [23]. Because the birds are thought to roost in caves or deep gorges only, and seeds that germinate in caves do not develop properly [17], [25], [26], the role of *Steatornis caripensis* as specialized dispersers has been questioned [25], [27].

We remotely studied the behaviour of *Steatornis caripensis*, in order to elucidate previously unknown aspects of this species' life history that could relate to the significance of its role in seed dispersal. In addition the study would provide information on the species land use around its cave roost site. This is of significance as the national park boundaries for that area were selected in order to protect this species. Using GPS/accelerometers that allow remote monitoring of the behaviour of an animal we will show how the application of modern observational ‘bio-logging’ techniques [12], [28] can quickly improve our understanding of a species' role in the ecosystem, with implications for ecology and conservation.

## Materials and Methods

Experiments on *Steatornis caripensis* were conducted under permits from the Ministerio del Ambiente (#2255) and Instituto Nacional de Parques (Inparques, #0789). We adhered to the AOU special committee recommendations for the use of wild birds in research. A Venezuelan National Park ranger accompanied our research team during work in the “Monumento Natural Alejandro de Humbolt”.

We studied *Steatornis caripensis* at the ‘Cueva del Guácharo’, or Humboldt Cave, in North-eastern Venezuela (10.1716°N, 63.5539°W). The cave is the site of the type specimen which Alexander von Humdoldt collected in 1799 during his “Journeys to the Aequinoctial Regions of the New World” [15]. The cave is known as perhaps the largest amassment of *Steatornis caripensis* and supposed to harbour as many as 20000 individuals at times [29]. The cave is within Parque Nacional el Guácharo, which encompasses approximately 50000 HA of protected mountain forests known to be used by *Steatornis caripensis*. We decided to track *Steatornis caripensis* in October 2007 toward the end of their chick rearing period [26]. We expected birds to be site faithful at this time, based on previous evidence [23].

Tags were deployed on the nights of 11/10/07 (2), 12/10/07 (4), 13/10/07 (2) and the morning of 15/10/07 (4). To catch oilbirds we put up one 12 m long, 4-shelf (2 meter) high mist net close to the entrance of the cave, approximately 30 minutes after sunset, when *Steatornis caripensis* were leaving the cave *en mass*. We then shut off all our lamps and waited for the birds to fly past/into the net. We repeated this procedure until we either had four birds caught or approximately 30 minutes had passed. We then lowered and removed the net, and left the cave immediately in order not to disturb the remainder of the birds.

Outside the cave, in the open, using head lamps, we weighed the birds to the nearest 10 g using spring balances and selected adult birds only, determined by examining plumage wear of their wing and tail feathers and by discarding individuals that had wing lengths below 300 mm, the asymptotic wing length (C. Bosque, pers. obs.). All birds weighed enough that the logger was between 5 and 6% of their bodyweight (average body mass 419.7±4.2 g). Given that during the breeding season birds must carry back up to 160 g of additional food for their young [30], it was unlikely that carrying this weight at this time (after chicks had fledged) would be a problem for the birds. We then immediately attached GPS/acceleration loggers. In six birds a harness around the breast, wings and legs was used, made of slightly stretchable nylon paracord. The 22 g logger sat nicely in the birds' back, approximately in the centre of gravity. In the other 6 birds a backpack glue-on system was used to test whether birds wearing the harness system would show different behaviour from those wearing a backpack. We found no obvious differences, all birds irrespective of logger attachment method flew fine, and thus we pooled all data for final analysis. When we released the birds (between 10 min to 1 h after capture), they all flew off well and joined the stream of other oilbirds leaving the cave towards their presumed foraging areas, or flew deep into the cave when captured and released in the morning.

To read out the data, we walked the entire length of the chamber once each day, for 17 days in total, during the late afternoon hours, holding a receiver base station in our hand. The GPS/acceleration loggers were programmed to contact the base station on a frequency of 868.3 MHz every 20 seconds. Whenever a birds was in the cave (as determined retrospectively from logged GPS data), we easily received and downloaded all its data in a simple walk through the cave.

The GPS/acceleration loggers are produced by e-obs GmbH (Munich, Germany) and feature programmable GPS logging and give-up times (important to conserve battery when a birds is in the cave without view of the GPS satellites). Ten of 12 loggers were programmed with 600 s intervals between GPS fixes and two with 900 s intervals. GPS was off between 10∶00 h and 22∶30 h in the 600 s interval loggers and between 9∶30 h and 22∶30 h in the 900 s loggers. This resulted in between 120 and 260 GPS locations per bird and allowed up to four nights recording. e-obs loggers also record 1, 2 or 3D acceleration, at programmable intervals, from about 3 Hz to 2000 Hz. Tags 19 and 20 recorded 747 bytes of data at 56.23 Hz every 300 s on one axis (up-down). Tags 21 and 29 recorded 747 bytes at 56.23 Hz every 120 s on two axes (up-down, back forward). Tags 8, 25, 28 and 30 recorded 747 bytes at 31.62 Hz every 120 s on one axis (up-down). Tags 22, 24, 26 and 27 recorded 747 bytes at 56.23 Hz every 120 s on two axes (up-down, back-forward). The battery life for download was much longer than the battery life for the final GPS fix so data could be retrieved at the cave even when GPS sampling had expired. The net download speed of the UHF radio link is about 1 MByte per min, via a simple base station that can additionally be outfitted with a high-gain directional antenna.

GPS points were plotted using Google Earth Pro^©^, which in combination with acceleration data analysed in e-obs visualisation tool allowed the determination of active flights to foraging locations or to a roosting site. Data was subsequently transferred to google maps^©^ for display. The e-obs visualisation tool also allowed the determination of wing beat frequency. Summary statistics were calculated from flights identified as these activities. Efficiency of flights was calculated by dividing the straight line distance between start and end point by the actual distance travelled. For statistical tests we used SPSS 15.0. We report means ± standard error if not otherwise noted. Data were entered into a beta-version of Movebank (www.movebank.org), a global repository of animal movement data.

## Results

### GPS Locations

**Figure 1:**
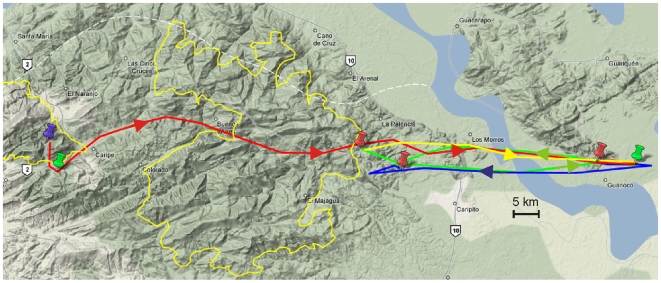
GPS locations for bird tagged with logger number 25. Roosting and foraging sites are indicated by markers. Green markers are foraging sites and red are roosting sites. The blue marker is the Cueva del Guácharo. Days of travel are indicated by colour: day 1, red, day 2, blue, day 3 green, day 4 yellow. The yellow lines mark the boundaries of both sectors of the Parque Nacional el Guácharo.

**Figure 2:**
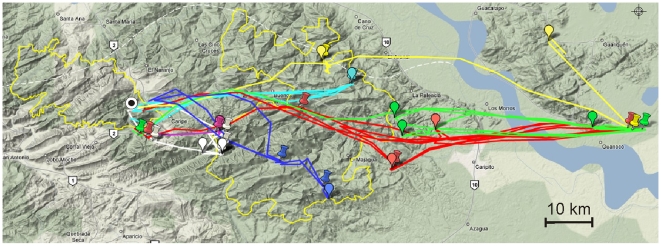
The tracks of all birds obtained during the study. Foraging and roosting sites used by *Steatornis caripensis* during the period of study, overlaid on the tracks obtained by the GPS. The drawing pin markers indicate foraging sites and the balloon markers indicate roosting sites, with the colour matching the birds track. The circular marker with the black dot is the Cueva del Guácharo. Birds are distinguished by different coloured tracks.

**Figure 3:**
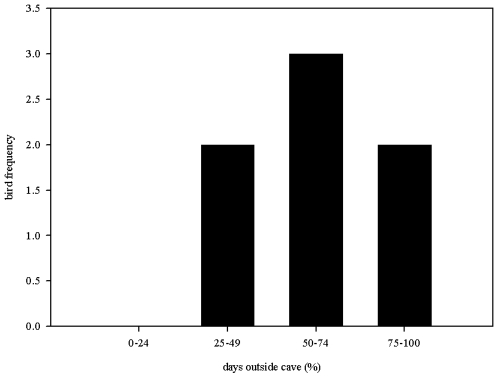
Percentage of nights spent roosting outside the Cueva del Guácharo during the period of data collection.

Eight of the 12 loggers deployed were downloaded at the cave before battery life expired. One bird (logger 27) did not leave the cave before the GPS battery expired and so only acceleration data were available from this bird. The mean distance of the furthest foraging site from the cave was 44.4±10.7 km with a maximum distance of 73.5 km, by the bird carrying logger 25 (Fig. 1). Roosting trees were 32.0±5.4 km away from caves. Most importantly for seed dispersal, the average distance from the last foraging tree to the roost tree was 10.0±4.6 km (Fig. 2), when roosting in the forest. GPS locations indicated that birds spent 66±8.0% of roosting time during the study outside the cave (Fig. 3). 57.1% of foraging sites and 59.4±17.0% of roosting sites fell inside the boundaries of current national park area placed to protect oilbirds and the pristine surrounding mountain forest (Fig. 2). Birds flew significantly faster when returning to a roost than when flying out to a foraging site (Wilcoxon matched pairs, n = 7, median out = 22.44, median return = 26.01, Z = −1.992, p = 0.046). There was however no difference in the efficiency of flights between outward and return journeys (Wilcoxon matched pairs, n = 7, median out = 0.96, median return = 0.97, Z = −0.507, p>0.05).

### Acceleration Data

**Figure 4:**
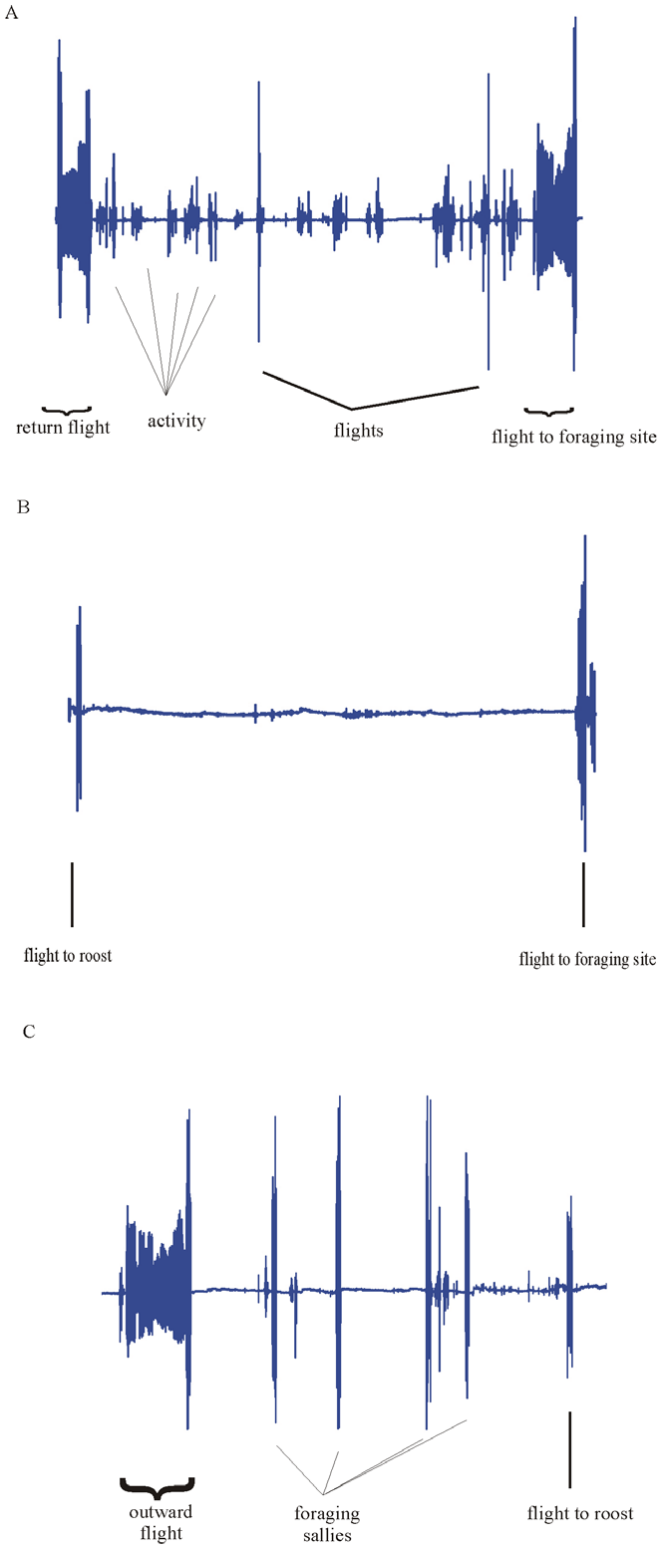
Acceleration trace of bird carrying tag 19. Recorded on a single (up-down) axis a) while roosting in the Cueva del Guácharo, b) while roosting in the forest and c) a trace of night time activity.

The acceleration data were analysed using the single up-down (Z) axis and indicate that during the sampled time birds spent an average of 32.8±4.1% of the sampled time active when roosting in the cave, but only 3.0±0.1% of the time active when roosting outside the cave (Fig. 4a, b)). Birds spent on average 25.1±5.2% of the sampled time during the night actively foraging, not including cruising flights to and from trees or the cave (Fig. 4c). The acceleration traces indicated no activity between these short foraging bouts.

The accelerometer allowed the wing beat frequency to be analysed during bouts of activity. Wing beat was significantly higher during foraging bouts than during either flights to a foraging site or flights to a roost (ANOVA: F_2,12_ = 36.394, p<0.0001, Bonferroni: out vs. return, p>0.05, out vs. forage, p<0.0001, return vs. forage, p<0.0001).

## Discussion

**Figure 5:**
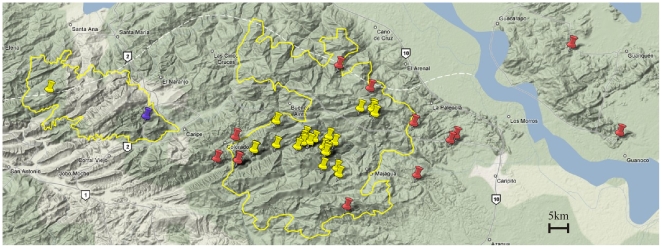
Roosting sites (red markers) of *Steatornis caripensis* plotted in relation to known roosting caves (yellow markers) in the region. The blue marker is the Cueva del Guácharo.

The data obtained here provide new insights into the behaviour of a unique nocturnal frugivore. Previous evidence suggested that *Steatornis caripensis* make foraging trips for fruit on a nightly basis, returning to the cave at the end of each night [17], [23]. Observations of high activity at foraging stands suggested that *Steatornis caripensis* would forage for fruit constantly throughout the night, breaking only to return to the cave [23]. Our data indicate that *Steatornis caripensis* do not continuously fly throughout the night and that individuals do not return to the Cueva nightly but make extended foraging trips over a number of nights. Whenever birds stay outside the cave for a few days they roost in trees in the forest during daylight hours. Plotting the position of known *Steatornis caripensis* caves indicates that roosting sites do not coincide with these (Fig. 5). Our data on diurnal behaviours of *Steatornis caripensis* confirm anecdotal reports of *Steatornis caripensis* roosting in trees [20], [21] and also supports the data from seed traps at the Cueva del Guácharo which suggest a drop in the number of seeds brought back to the cave at this time of year [25]. Our data also indicate that roosting sites in the forest are not the same place that the birds forage, which also indicates that they are effective seed dispersers. This has major implications for the status of oilbirds within their ecosystem. By staying out near, but not at foraging sites for several nights increases their effectiveness as seed dispersers. The birds foraged up to 75 km from their roost, the Cueva del Guácharo; similar to data from radio tracking which suggested that they may forage 120 km from the Cueva nightly [23]. This distance is also beyond the boundary of the national park put in place to protect these animals. Approximately 40% of roosting and foraging sites were outside the boundaries of the national park. The high efficiency of the flights and the higher speed with which birds flew to roosting sites than to foraging sites suggests that the roosting sites may have been familiar to them, there was no apparent search pattern displayed in the tracks.

The addition of accelerometer data to the GPS data allowed us to make additional inferences about the behaviour of the animals. When roosting in the forest during the day, the data indicate that the birds remained inactive, making few if any movements during daylight hours. When roosting in the cave however, the birds maintained a significantly higher level of activity. Although Cueva del Guácharo is a tourist attraction and so the possibility of human disturbance exists, our data indicated that this high activity was maintained even on days when tourists were not allowed into the cave. Roosting in the cave therefore brings the high cost of activity compared to roosting in the forest, where the animal remains motionless all day. The cave roosting behaviour of *Steatornis caripensis* therefore seems to be a trade off between the benefits of avoiding diurnal predators and the high cost of remaining active, possibly to defend roosting and nesting ledges or establish dominance hierarchies, resulting in a partitioning of roosting between the cave and open forest. In the breeding season birds are forced to spend more time in the cave as chicks less than 250 g in weight are attended for 84% of the time [30]. It is likely therefore that at this time pairs share nights on the nest with the other partner foraging out and roosting in the forest for a number of nights, but further study is needed to determine if this is indeed the case.

The accelerometer data also indicated that contrary to the previous assertion of [23], birds do not forage constantly throughout the night but in fact only spend approximately 25% of the night foraging. The rest of the time is spent inactive. Wing beat frequency indicates a higher frequency when foraging than during cruising flights to or from foraging sites. This is most likely because the birds briefly hover while taking fruit from the trees.

The new remotely downloadable GPS with accelerometer used here has given new insights into the behaviour of *Steatornis caripensis*, a unique frugivorous bird. It has indicated that the daily activity pattern of this animal is different from that assumed from previous observation. The pattern of foraging shown by *Steatornis caripensis* indicates that it is a far more effective seed disperser than was previously thought [25]. The combination of high resolution GPS and accelerometer data allows the remote monitoring of the behaviour of a wild bird and has revealed previously unknown aspects of its daily activity patterns. It has been proposed that predation pressure on nests was the major selective force leading to cave breeding and roosting [20]. The indication that they spend only a percentage of their time in the cave raises new questions about the trade offs of colony living in confined spaces. The ability to remotely monitor the behaviour of highly mobile animals in detail may prove that many assumptions that are held about such species, even well studies ones, to be unfounded. With the constant development of new technology to study such animals, the next decade may bring a new golden age of discovery of wild animals such as *Steatornis caripensis*.
